# Barcode ITS2: a useful tool for identifying *Trachelospermum jasminoides* and a good monitor for medicine market

**DOI:** 10.1038/s41598-017-04674-w

**Published:** 2017-07-11

**Authors:** Ning Yu, Yu-long Wei, Xin Zhang, Ning Zhu, Yan-li Wang, Yue Zhu, Hai-ping Zhang, Fen-mei Li, Lan Yang, Jia-qi Sun, Ai-dong Sun

**Affiliations:** 1 College of Biological Sciences and Technology, Beijing Forestry University, Beijing, 100083 China; 2 Beijing Key Laboratory of Forest Food Processing and Safety, Beijing Forestry University, Beijing, 100083 China

## Abstract

*Trachelospermum jasminoides* is commonly used in traditional Chinese medicine. However, the use of the plant’s local alternatives is frequent, causing potential clinical problems. The *T*. *jasminoides* sold in the medicine market is commonly dried and sliced, making traditional identification methods difficult. In this study, the ITS2 region was evaluated on 127 sequences representing *T*. *jasminoides* and its local alternatives according to PCR and sequencing rates, intra- and inter-specific divergences, secondary structure, and discrimination capacity. Results indicated the 100% success rates of PCR and sequencing and the obvious presence of a barcoding gap. Results of BLAST 1, nearest distance and neighbor-joining tree methods showed that barcode ITS2 could successfully identify all the texted samples. The secondary structures of the ITS2 region provided another dimensionality for species identification. Two-dimensional images were obtained for better and easier identification. Previous studies on DNA barcoding concentrated more on the same family, genus, or species. However, an ideal barcode should be variable enough to identify closely related species. Meanwhile, the barcodes should also be conservative in identifying distantly related species. This study highlights the application of barcode ITS2 in solving practical problems in the distantly related local alternatives of medical plants.

## Introduction

*Trachelospermum jasminoides* (Apocynaceae), a medicinal vine plant known as “luoshiteng”, is widely used in traditional Chinese medicine^1, 2^. *T*. *jasminoides*, which are found mostly in Henan, Shandong, Anhui, Jiangsu, Zhejiang, and Fujian provinces of China, commonly grows around trees or climbs rock walls. According to the Chinese medicine theory, *T*. *jasminoides* can be used for the treatment of cool blood swelling, rheumatic fever, spastic muscles, waist and knee pain, sore throat, swelling, and traumatic injury^3^. Moreover, the seeds of *T*. *jasminoides* are hemostatic and cardiotonic^4^. The leaves and stems of *T*. *jasminoides* have been found to contain several kinds of compounds, including flavonoids, lignans, sterols, and tri-terpenoids. Therefore, the plant possesses pharmacological activities, including anti-inflammatory, anti-bacterial, and anti-viral functions^5–7^. Given these properties, *T*. *jasminoides* has been used heavily in clinical treatments and has attracted increasing attention in the field of medicine.

However, some *T*. *jasminoides* alternatives are frequently used as local medicinal plants in many places because of geographical and historical factors, causing potential medical care problems. For example, *Ficus tikoua* (Moraceae), a Miao native mainly produced in south China, usually substitutes for *T*. *jasminoides* in parts of the Guizhou province. *Ficus pumila* (Moraceae), another medicinal plant with similar plant morphology and living environment to *T*. *jasminoides*, is usually substituted for *T*. *jasminoides* in northeast, north, and east China. Moreover, *Euonymus fortunei* is described as *T*. *jasminoides* and commonly substitutes for *T*. *jasminoides* in clinical applications in the Jiangsu, Hubei, Shanxi, Shandong, and Henan provinces. The alternatives share the same name and several clinical functions with *T*. *jasminoides*, such as the subsidence of swelling, rheumatic pain, and traumatic injury. However, their functions and curative effects are not exactly the same because of their various compositions^8–11^. The inappropriate application of *T*. *jasminoides* may affect its medical efficacy. Therefore, the accurate identification of *T*. *jasminoides* is especially important. However, the *T*. *jasminoides* sold in the medicine market is commonly dried, sliced, or shredded, making the application of traditional identification methods more difficult. An accurate and fast tool is required, which calls to mind DNA barcoding.

DNA barcoding is a milestone in taxonomy in its use of short sequences from standard genome regions to identify species^12–14^. Unlike animal barcoding, the standard mitochondrial cytochrome *c* oxidase 1, DNA barcoding in plants has experienced a long time since it was first proposed because of its lower mutation rate and very little variation in the typically used plastid phylogenetic markers^15, 16^. Several DNA regions, including *atpF–atpH*, *matK*, *rbcL*, *ndhj*, *ycf5*, *accD*, *rpoB*, *rpoC1*, *psbK–psbI*, *trnH–psbA*, *trnL-F*, and ITS as well as their combinations, have been advocated to provide a standard plant barcode^17–23^. Internal transcribed spacer 2 (ITS2), a part of the nuclear rDNA, has been proposed as a candidate DNA barcode^24, 25^ and has attracted increasing attention in the recent years. The ITS2 region is an ideal barcode because of its short length, easy amplification with a single pair of primers, high sequencing efficiency, and high variation between species^26, 27^. Moreover, molecular morphological characteristics based on the secondary structures of ITS2 provide another identifiable ground for species and better discrimination^28, 29^. Many research indicated that ITS2 exhibits a good capacity for species identification^30, 31^. Furthermore, the ITS2 region has been used to supervise the proportions and varieties of adulterant species^32, 33^.

Previous studies on DNA barcoding concentrated more on the same family, genus, or species. However, the substitution or adulteration of medical plants are not generally closely related species^34, 35^. An ideal barcode should be variable enough to identify closely related species while being conservative in identifying distantly related species. This study highlights the application of barcode ITS2 in solving practical problems in the distantly related local alternatives of medicinal plants. The ITS2 region was used to barcode 127 sequences, including those of *T*. *jasminoides* and its local alternatives. Meanwhile, the adulterant proportions of *T*. *jasminoides* in the medicine market was investigated using barcode ITS2.

## Results

### Amplification, sequencing, and alignment

Genomic DNA was extracted from the 101 samples. Electrophoretic results indicated the six bright bands of leaf extractions, whereas the other bands of dried samples collected from markets or drugstores were smeared or invisible. In these cases, the amplification success rate of the ITS2 regions for all the 101 samples were 100%. All the PCR products of the ITS2 barcode were successfully sequenced, and all the bidirectional sequences were of high quality. Before alignment, the lengths of the ITS2 sequences of *T*. *jasminoides*, *F*. *pumila*, *F*. *tikoua*, and *E*. *fortunei* were 222, 239, 241, and 220 bp, respectively. The G  +  C contents ranged from 61.3% to 74.1%, of T. *jasminoides*, *F*. *pumila*, *F*. *tikoua*, *and E*. *fortunei* was 61.3, 71.1, 67.2, and 74.1%, respectively. The aligned length was 270 bp, and 126 variable sites existed with a rate of 46.7% (Table 1). Therefore, the ITS2 sequences for the collected species were relatively variable.

**Table 1:** Sequence information of samples.

	ITS2
Amplification efficiency (%)	100
Sequencing efficiency (%)	100
Length of all taxa (bp)	220–241
Aligned length (bp)	270
G+C content range in all taxa (%)	61.3–74.1
Number (and %) of variable sites in all taxa	126 (46.7%)

### Genetic divergence with and between species

All the 127 ITS2 sequences were used to calculate the K2P genetic distances. In the present study, only one haplotype of *T*. *jasminoides* species was found, no variable sites were observed, and the intraspecific distance of *T*. *jasminoides* was 0.000. The interspecific distance between *T*. *jasminoides* and its local alternatives varied from 0.488 to 0.607. The minimum interspecific distance of 0.488 between *T*. *jasminoides* and *F*. *pumila* was far larger than the maximum intraspecific distance (Table 2).

**Table 2:** Analysis of intra-specific variation and inter-specific divergence of the ITS2 sequences.

K2P genetic distances	Genetic distances
Intra-specific distance of *T*. *jasminoides*	0.000
Inter-specific distance between *T*. *jasminoides* and *E*. *fortunei*	0.567–0.607
Inter-specific distance between *T*. *jasminoides* and *F*. *tikoua*	0.505–0.522
Inter-specific distance between *T*. *jasminoides* and *F*. *pumila*	0.488–0.502

### Barcoding gap assessment

**Figure 1:**
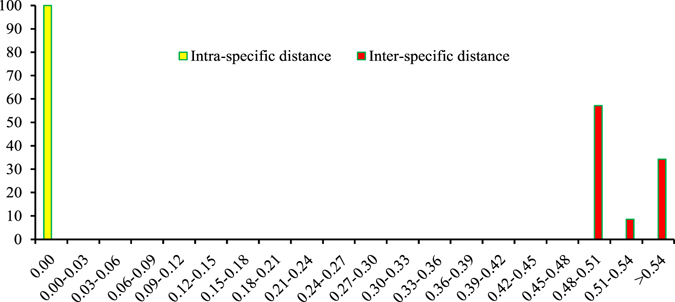
Relative distribution of interspecific divergence between congeneric species and intraspecific distances for ITS2 locus.

Barcoding gap is an important index in determining whether a DNA barcode is suitable or not. In the present study, the distributions of intra- and inter-specific divergences in ITS2 barcode were examined at a scale bar of 0.03 distance units. The intra-specific distance of the ITS2 region was low because only one haplotype of the *T*. *jasminoides* species was present. The ITS2 region presented a very high inter-specific variation and an obvious barcoding gap was noted because the inter-specific variation never overlapped with the intra-specific distance (Fig. 1). Results indicate that the ITS2 region was variable and consistent with the high inter-specific divergence in medicinal plants^24^.

### Species identification efficiency

The identification efficiency of the ITS2 region was evaluated by the BLAST1 and nearest distance methods. In this study, results showed that ITS2 region could successfully identify all the samples with both methods without incorrect or ambiguous identification (Table 3).

**Table 3:** The identification efficiency of the ITS2 sequences using different methods.

Marker	Method of species identification	Number of samples	Correct identification	Incorrect identification	Ambiguous identification
ITS2	BLAST 1	101	100%	0	0
	Nearest distance	101	100%	0	0

### Analysis of secondary structure

**Figure 2:**
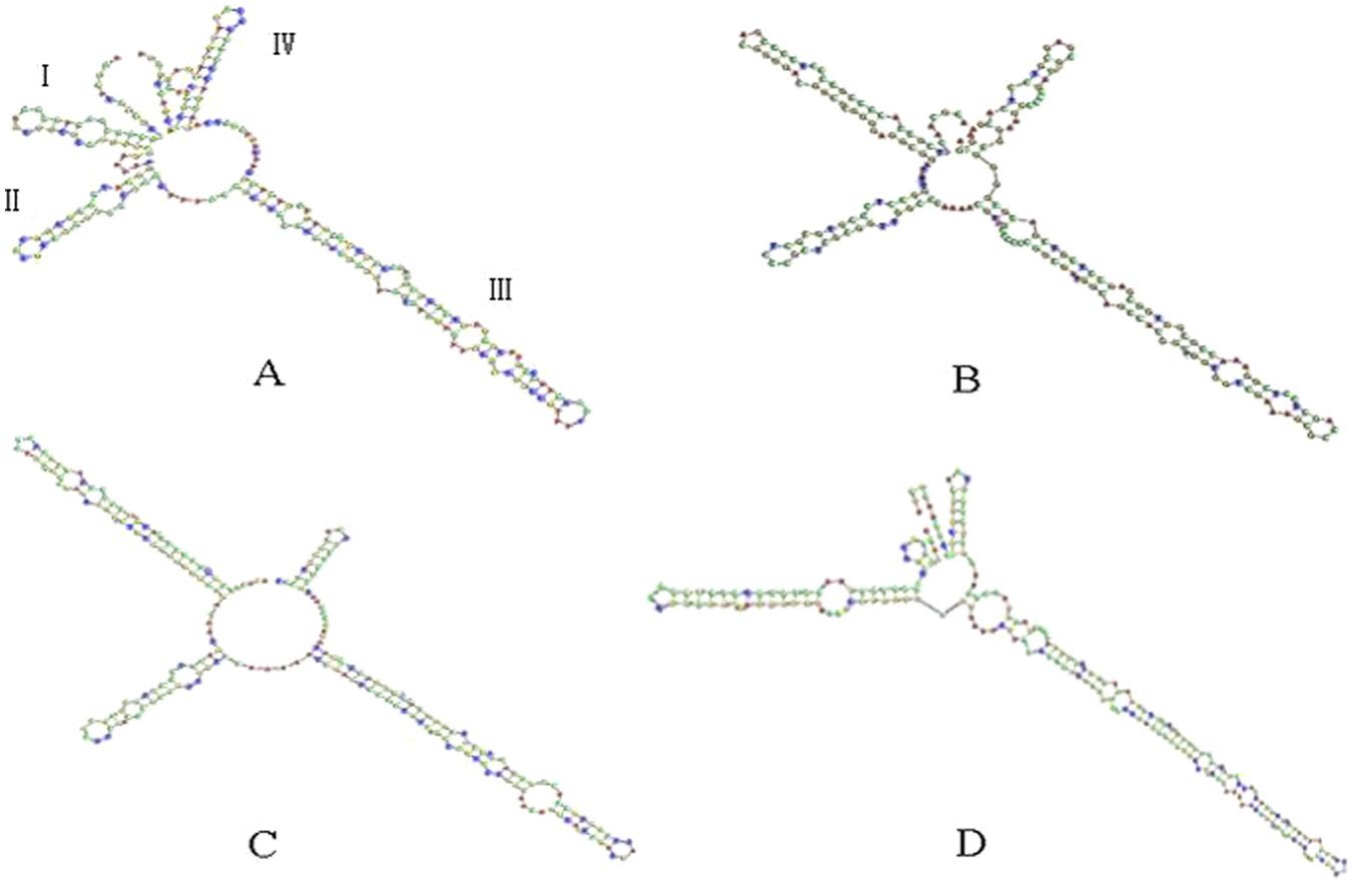
Secondary structure of ITS2 in *T*. *jasminoides* and its adulterants. (**A**) *T*. *jasminoides* (**B**) *E*. *fortunei* (**C**) *F*. *tikoua* (**D**) *F*. *pumila*.

In this study, the secondary structures of ITS2 regions were predicted to identify the species. A central ring and four similar helices, namely, Helix I, II, III, and IV, existed in the ITS2 secondary structures of *T*. *jasminoides* and its common local adulterants. However, the secondary structures of ITS2 among these species displayed significant differences in the four helices in terms of stem loop number, size, position, and degree of angles from the center of the spiral arm (Fig. 2). Thus, the secondary structure of ITS2 provides another dimensionality for species identification at the molecular morphological characteristics level.

### NJ tree analysis

**Figure 3:**
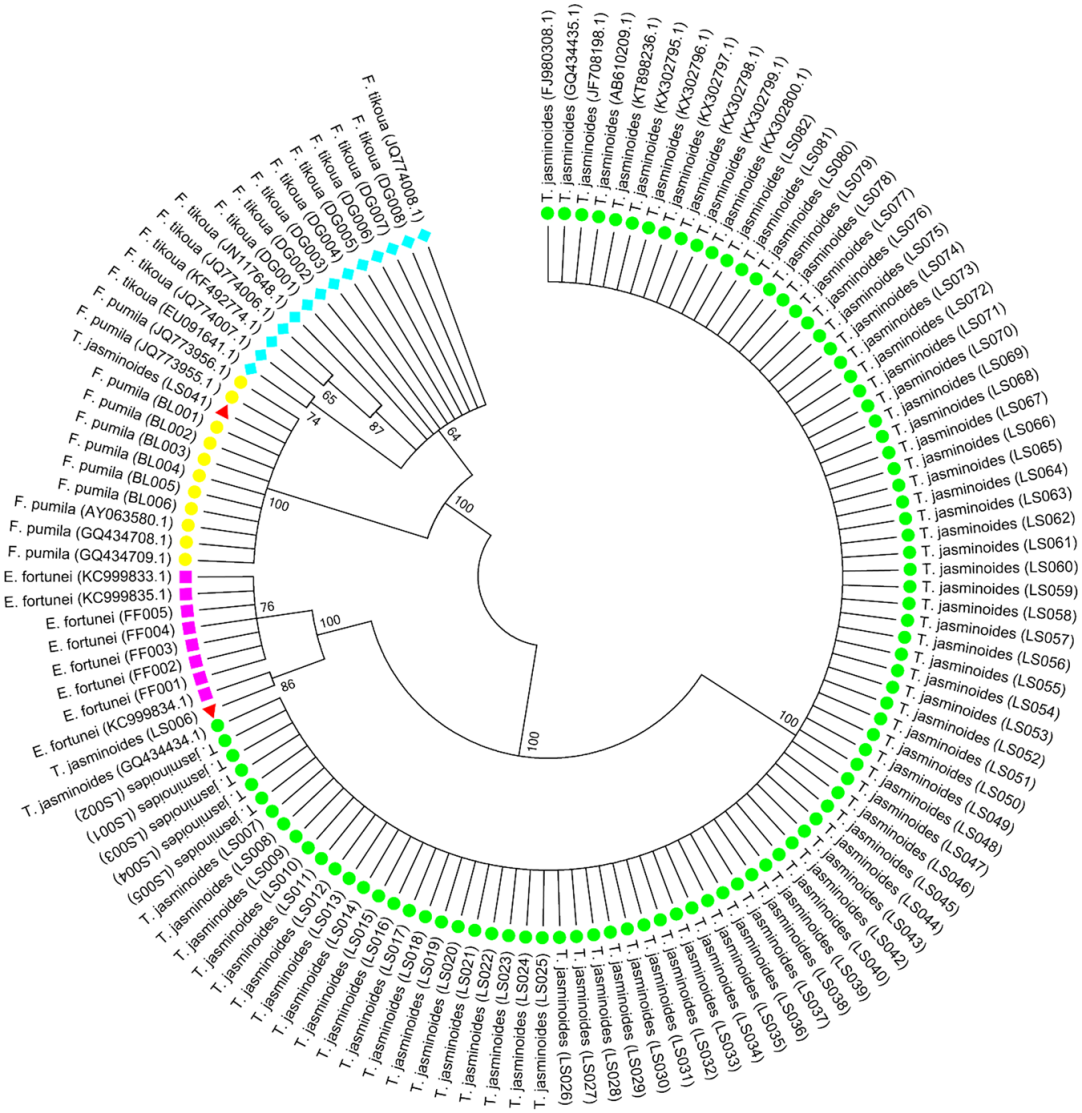
NJ tree of *T. jasminoides* and its adulterants constructed with ITS2 sequences. The 1000 replicates bootstrap scores are shown (≥60) for each branch.

An NJ tree was constructed using all the 127 ITS2 sequences. Results demonstrated that all 92 *T*. *jasminoides* species clustered into one clade, while *F*. *pumila*, *F*. *tikoua*, and *E*. *fortunei* clustered into their own clades. Notably, LS006 and LS041 samples were identified as *E*. *fortunei* and *F*. *pumila*, respectively, indicating that adulterants of *T*. *jasminoides* existed in the medicine market and ITS2 can be used as a good monitor (Fig. 3). Overall, NJ tree can clearly distinguish between *T*. *jasminoides* and its common local adulterants.

### Two-dimensional DNA barcoding

**Figure 4:**
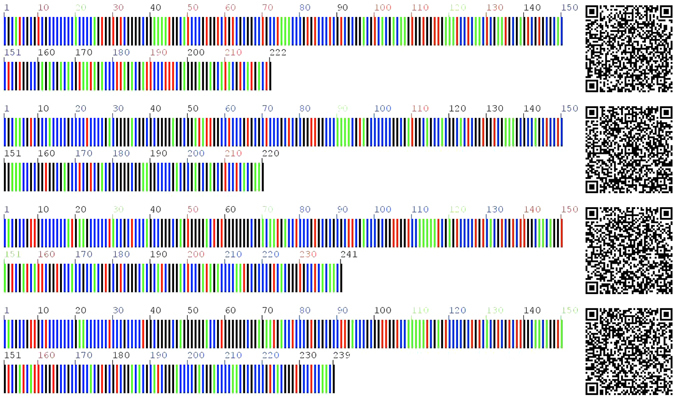
DNA barcoding and two-dimensional DNA barcoding image of ITS2 sequences ( A  T C  G *T*. *jasminoides* 222 bp; *E*. *fortunei* 220 bp; *F*. *pumila* 239 bp; *F*. *tikoua* 241 bp).

In the current stage, “DNA barcode” only represents DNA sequences that are unamenable to information storage, recognition, and retrieval. The QR code was found superior in representing DNA barcode sequences efficiently^36^. The ITS2 sequences of *T*. *jasminoides* and its local adulterants were transformed into QR codes and two-dimensional DNA barcoding images were used in this study (Fig. 4). In the left colored DNA image, the different colors represent different nucleotides and the numbers represent the lengths of the sequences, which can be used in obtaining clear sequence information. By scanning the two-dimensional code at the right with the scanner (e.g., mobile terminal), the species sequence can be obtained. After sending the sequences to the database for identification, results are returned to the scanner, making the identification more convenient and rapid.

## Discussion

### Barcode ITS2: a good tool for identification

The substitution of medical herbs with local alternatives in clinical treatment is prevalent. Therefore, a rapid and accurate identification method of medical herbs is urgently needed. DNA barcoding is not influenced by external factors, biological development stage, or organ tissue, thus providing a basis for identification at the gene level. ITS2 region was proposed as the standard marker for medical plant species identification and for phylogenetic analysis^37, 38^. In this study, the ITS2 sequence was used to distinguish *T*. *jasminoides* from distantly related local alternatives with one universal pair of primer for PCR amplification. DNA was isolated from dried samples, which were degraded on several levels. For dried stem samples, tissues with more living cells, appropriate increase of sample quantity and extended water bath time will be better for DNA extraction. The success rate was 100% because ITS2 is a multi-copy region, and amplifying the degraded DNA was easier than the other regions. In the present study, high-quality bidirectional sequences were obtained for 100% of the ITS2 region without manually editing the trace files. The ITS2 sequence length ranged from 220 bp to 241 bp, satisfying the short length criterion of good barcoding. All the ITS2 sequences were used to perform BLAST, and results indicated the absence of fungal contamination in the samples. Barcoding gap is an important criterion for an ideal DNA barcode^39, 40^. The ITS2 region exhibited conservative intra-specific divergence and high inter-specific variation, and the barcoding gap was obvious. In addition, the secondary structures of the ITS2 region provided morphological characteristics that can help improve species identification^28, 41^. The secondary structures of *T*. *jasminoides* and its local alternatives displayed different stem loop numbers, positions, sizes, and angle degrees. Discriminatory power is another criterion for a good DNA barcode, and the results of the BLAST 1 and nearest distance methods indicated that ITS2 regions successfully identified all the texted samples. Furthermore, according to the NJ tree results, *T*. *jasminoides* and its local alternatives clustered into their own clades. Therefore, barcode ITS2 successfully identified *T*. *jasminoides* and its local alternatives and exhibit an ideal barcode criterion. Our results corresponded well with the results of previous studies^42, 43^.

Notably, the internal transcribed spacer of nuclear DNA region exist multiple copies within each cell, it is doubtful whether the PCR sequences would be stable and representative, which making the use of ITS2 barcoding more complicated^14, 56^. Song *et al*. suggested that major variants of the ITS2 region is sufficient for phylogenetic analysis and species identification in most cases^31^. The current study indicated that the multi-copy of the ITS2 sequences is not a problem in the identification of *T*. *jasminoides* and its adulterants in this study, which highlights the universality of the ITS2 region as a DNA barcode. To our knowledge, this is the first time that *T*. *jasminoides* and its adulterants have been identified using the ITS2 region in such a large sample size. In addition, each species were represented by more three samples for better determination of intraspecific variation. Moreover, in previous studies, most of the samples were fresh or silica gel dried, while 93.5% of the samples were dried and sliced in the current study, which broaden the practical application of the ITS2 region in the herbal plant field.

### DNA barcoding for medicine market supervision

The global trade of raw drugs has been witnessed in the past decades, and herbal product market has become much more promising. However, an increase in immoral commercial practices has emerged to make more profits, whereby authentic herbs are substituted with cheap, less effective, and often deleterious herbs. Accurate and fast species identification is the key to herbal market safety^44^. Traditional methods commonly require professionals, such as taxonomists, who require significant amount of time and are sometimes inconsistent in their opinions on identification at the industrial level. DNA is more stable than other macromolecules (protein and RNA), and genetic molecule is not affected by external factors and is easily isolated in all tissues^45^. Many research have been conducted using DNA barcoding to supervise the medicine market^46, 47^. The present study broadens the use of ITS2 barcode to market products. Two adulterants were detected, including one *F*. *pumila* and one *E*. *fortunei*, from the 79 dried *T*. *jasminoides* samples purchased from drug stores and herbal markets. Barcode ITS2 was proposed for application in monitoring *T*. *jasminoides* in the medicine market. Similarly, DNA barcoding should be embraced for identifying herbal products through the testing of raw materials applied in the herbal industry^48^.

### DNA barcoding in two-dimension

DNA barcoding is the final product of a handled DNA barcoder, which contains components for DNA extraction, amplification, and sequencing and a DNA barcode analysis engine with the associated software tools and database. To realize the “life barcoder” better, an appropriate format is essential. At present, “DNA barcode” specifically refers to DNA sequences. However, the large printout size and difficulty in information retrieval of the sequences limit barcoding in practical applications. Therefore, a new format of retrieving DNA barcode information is urgently required. Barcode technology has been applied in the manufacturing and retailing industries for a couple of decades. This well-developed technology has been used to investigate the symbology that could represent DNA barcode sequences better, as reported by Liu *et al*.^36^. Their results indicated that the QR code had the largest coding capacity and relatively high compression ratio. In the present study, the ITS2 sequences of *T*. *jasminoides* and its local alternatives were converted to QR codes, and two-dimensional images were obtained along with the clear sequence information. The assurance of the genuine origin of herbal materials is crucial, two-dimensional DNA barcoding can monitor the source of medicinal materials from the origin. The true and false recognition of the herbal materials can be performed in the field, as well as the random inspection using two-dimensional DNA barcoding. QR code-based DNA barcodes promote DNA barcoding applications to a more practical level and extensively illustrate the potential purpose of DNA barcoding in the identification of medicinal plants to ensure product safety. At present, two-dimensional DNA barcoding merely encompasses Latin names and sequence information for species, pictures of medicinal herbs and property descriptions should be included for better identification.

### Challenge and promise for DNA barcoding

In the present study, barcode ITS2 exhibited a powerful discrimination capacity toward *T*. *jasminoides* and its local alternatives, perfectly resolving practical problems. However, the ITS2 barcode is not sufficient to identify the heavily processed materials with degraded DNA, which are difficult to amplify. A nucleotide signature specific to the tested sample within the ITS2 region is also an effective approach. The ideal nucleotide signature should be completely conserved within a specific species^49^. In addition, whether the genuine *T*. *jasminoides* samples texted in this study satisfy the quality of Chinese pharmacopoeia standard, in which the content of tracheloside must be more than 0.45%, require further study. Moreover, attention should be given to the possibility that herbal products may not only be contaminated at the plant species level. The substitution of the non-prescribed plant part or a prescribed part harvested during the wrong season also present different medical effects. Thus, DNA barcoding will fail to identify lower quality products under this circumstance^44^. Therefore, merely relying on DNA barcoding to perform quality control for medical plants is insufficient. DNA barcoding should be supplemented with morphological and biochemical traits for raw drug supervision to guarantee clinical safety^50^.

## Methods

### Sampling

A total of 101 samples were collected from the Hebei, Henan, Hubei, Hunan, Shanxi, Sichuan, Anhui, Shandong, Liaoning, Jilin, Heilongjiang, Xiamen, and Guangxi provinces and Beijing municipality. Among them, 82 samples were *T*. *jasminoides* (79 stem and 3 leaf samples), six were *F*. *pumila* (5 stem and 1 leaf samples), eight were *F*. *tikoua* (7 stem and 1 leaf samples), and five were *E*. *fortunei* (4 stem and 1 leaf samples). The collected data are listed in Appendix S1. The leaf samples collected from the field were silica gel-dried, while the stem samples purchased in the medicine markets and drugstores were already dried. All corresponding voucher samples were deposited in the Beijing Forestry University, Beijing, China. In addition, 12 published *T*. *jasminoide*s, *5* *F*. *pumila*, *6* *F*. *tikoua*, *and 3 E*. *fortunei* ITS2 sequences (or containing ITS2 sequence) were downloaded from the GenBank (Appendix S1).

### DNA isolation, amplification, and sequencing

The DNA extraction and amplification of the ITS2 locus of the 101 samples were conducted in the Laboratory of Molecular Biology at the Department of Biology. The samples were first scraped and then wiped with 75% ethanol to prevent fungal contamination. Then, 40 mg of the samples were rubbed with liquid nitrogen until they became powder. Total genomic DNA was isolated using a plant genomic DNA kit (Tiangen, China), which is based on the CTAB approach. The ITS2 sequences were amplified using a pair of universal primers: ITS2-2F, 5′-ATGCGATACTTGGTGTGAAT-3′ and ITS2-3R, 5′-GACGCTTCTCCAGACTACAAT-3′^24^. The primer pair was synthesized by the Shanghai Sangon Biotech and Service, Beijing branch (Beijing, China). PCR amplifications were performed in 25 µL reaction volumes containing 12.5 µL of 2  ×  EasyTaq PCR SuperMix (Beijing Baierdi Biothch Co., China), 8.5 µL of molecular grade water, 1 µL of each primer (2.5 µM), and 2 µL of the DNA template. The PCR reactions proceeded at 94 °C for 5 min, followed by 40 cycles at 94 °C for 45 s, 56 °C for 45 s, 72 °C for 1.5 min, and a final extension step at 72 °C for 10 min. The PCR products were sequenced in both directions by the Institute of Crop Science, Chinese Academy of Agricultural Sciences (Beijing, China).

### Data analysis

The raw trace files were trimmed and assembled using CodonCode Aligner 6.0.2 (CodonCode Co., USA). All ITS2 regions were annotated using the Hidden Markov model to remove the 5.8 S and 28 S sections^51^. Sequences with lengths less than 100 bp were eliminated as well as the sequences contaminated by fungi or other unnamed species^52^. The effectiveness of the ITS2 locus was evaluated with the following methods.

### Intra- and inter-specific divergences

All the 127 sequences were analyzed by MEGA 5.2.2^53^ to obtain the aligned length, G  +  C content range, and the number of variable sites. The sequences were aligned using Muscle, and the intra- and inter-species genetic distances were computed with the kimura-2-parameter (K2P) model in MEGA 5.2.2. Intra- and inter-species pairwise divergences were calculated as the barcoding gaps using TAXON DNA^54^.

### Authentication efficacy evaluation

Two identification methods, BLAST1 and nearest distance, were used to evaluate the authentication efficacy of the ITS2 region^55, 56^. All ITS2 sequences were regarded as query sequences, and BLAST program was performed in the BLAST 1 method. Three situations were considered. One is when the best BLAST hit of the query sequence is from the expected species, it is considered a correct identification. Another is when the best BLAST hits for a query sequence are from several species, including that of the expected species, they are regarded as an ambiguous identification. The other is when the best BLAST hit is not from the expected species, it is considered an incorrect identification. The nearest distance method is based on the smallest genetic distances, and correct identification indicates that the hit comes from the same species as that of the query. Ambiguous identification means that several hits have the same smallest genetic distances in the query sequence, and incorrect identification indicates that the hit is not from the query sequence.

### Tree-based method

A phylogenetic analysis of all the 127 sequences was performed using neighbor-joining (NJ) tree method to evaluate the identification capacity of the ITS2 locus, and node support was assessed based on 1000-replicate bootstrap tests. The identification of species with multiple individuals clustering into one clade based on the NJ tree method was considered successful when the bootstrap value was above 60%^57^.

### Secondary structure prediction

The deep level phylogeny derived from the ITS2 data largely agreed with the phylogenetic hypotheses from morphologic and other molecular evidence. The secondary structure of all the ITS2 sequences was predicted using the ITS2 Workbench (http://its2.bioapps.biozentrum.uni-wuerzburg.de/)^58^.

### Two-dimensional DNA barcoding

Quick response (QR) code was found to be a suitable symbology for the DNA barcode sequences. Based on the QR Code coding approach, the ITS2 sequences were transformed into two-dimensional images (http://qrfordna.dnsalias.org)^36^.

## Electronic supplementary material

Supplementary Information

## References

[1] Schuhfried E (2017). Withering of plucked *Trachelospermum jasminoides* (star jasmine) flowers-Time-dependent volatile compound profile obtained with SPME/GC-MS and proton transfer reaction-mass spectrometry (PTR-MS). Postharvest Biology and Technology..

[2] State Pharmacopoeia Committee. Pharmacopoeia of the People’s Republic of China Part I [253] (China Medical Science Press, Beijing, 2010).

[3] Song YC (2005). Characterization of graphislactone A as the antioxidant and free radical-scavenging substance from the culture of *Cephalosporium* sp. IFB-E001, an endophytic fungus in *Trachelospermum jasminoides*. Biological and Pharmaceutical Bulletin..

[4] Fatima T (1987). Indole alkaloids from *Trachelospermum jasminoides*. Planta medica..

[5] Sheu MJ (2009). Analgesic and anti-inflammatory activities of a water extract of *Trachelospermum jasminoides* (Apocynaceae). Journal of ethnopharmacology..

[6] Tan XQ (2010). Chemical constituents of *Trachelospermum jasminoides*. Natural product research..

[7] Jing L (2012). Trace chemical constituents contained in *Trachelospermum jasminoides* and structure identification. China journal of Chinese materia medica..

[8] Guan YX (2007). Chemical constituents in *Ficus tikoua* of Miao nationality. *Chinese Traditional and Herbal*. Drugs..

[9] Leong CN, Tako M, Hanashiro I, Tamaki H (2008). Antioxidant flavonoid glycosides from the leaves of *Ficus pumila* L. Food Chemistry..

[10] Ragasa CY (1999). A triterpene from *Ficus pumila*. Journal of Asian natural products research.

[11] Lai H, Huang X, Wei R (2009). Study on the extraction of the antioxidant component in *Euonymus fortunei* by two steps of release and hot-extraction. Applied Chemical Industry.

[12] Hebert PD, Cywinska A, Ball SL (2003). Biological identifications through DNA barcodes. *Proceedings of the Royal Society of London*. Series B: Biological Sciences.

[13] Marshall E, Will DNA (2005). bar codes breathe life into classification. Science..

[14] Hollingsworth PM (2011). Refining the DNA barcode for land plants. Proceedings of the National Academy of Sciences of the United States of America..

[15] Chase MW (2005). Land plants and DNA barcodes: short-term and long-term goals. Philosophical Transactions of the Royal Society B: Biological Sciences..

[16] Kress WJ (2005). Use of DNA barcodes to identify flowering plants. Proceedings of the National Academy of Sciences of the United States of America..

[17] Chase MW (2007). A proposal for a standardised protocol to barcode all land plants. Taxon..

[18] CBOL Plant Working Group. A (2009). DNA barcode for land plants. Proceedings of the National Academy of Sciences of the United States of America..

[19] Kress WJ (2009). Plant DNA barcodes and a community phylogeny of a tropical forest dynamics plot in Panama. Proceedings of the National Academy of Sciences of the United States of America..

[20] Larranaga N, Hormaza JI (2015). DNA barcoding of perennial fruit tree species of agronomic interest in the genus *Annona* (Annonaceae). Frontiers in plant science..

[21] Lahaye R (2008). DNA barcoding the floras of biodiversity hotspots. Proceedings of the National Academy of Sciences of the United States of America..

[22] Purushothaman N (2014). A tiered barcode authentication tool to differentiate medicinal Cassia species in India. Genetics and molecular research..

[23] Yu N (2016). Suitable DNA Barcoding for Identification and Supervision of *Piper kadsura* in Chinese Medicine Markets. Molecules..

[24] Chen S (2010). Validation of the ITS2 region as a novel DNA barcode for identifying medicinal plant species. PloS One..

[25] Yao H (2010). Use of ITS2 region as the universal DNA barcode for plants and animals. PloS One..

[26] Chiou SJ (2007). Authentication of medicinal herbs using PCR-amplified ITS2 with specific primers. Planta medica..

[27] China Plant BOL Group (2011). Comparative analysis of a large dataset indicates that internal transcribed spacer (ITS) should be incorporated into the core barcode for seed plants. Proceedings of the National Academy of Sciences of the United States of America..

[28] Grajales A, Aguilar C, Sánchez JA (2007). Phylogenetic reconstruction using secondary structures of Internal Transcribed Spacer 2 (ITS2, rDNA): finding the molecular and morphological gap in Caribbean gorgonian corals. BMC Evolutionary Biology..

[29] Gu W (2013). Application of the ITS2 region for barcoding medicinal plants of Selaginellaceae in Pteridophyta. PLoS One..

[30] Pang X (2011). Applying plant DNA barcodes for Rosaceae species identification. Cladistics..

[31] Song J (2012). Extensive pyrosequencing reveals frequent intra-genomic variations of internal transcribed spacer regions of nuclear ribosomal DNA. PloS One..

[32] Zhao S (2015). Internal transcribed spacer 2 barcode: a good tool for identifying *Acanthopanacis cortex*. Frontiers in plant science..

[33] Xin T (2015). Survey of commercial *Rhodiola* products revealed species diversity and potential safety issues. Scientific reports..

[34] Devaiah KM, Venkatasubramanian P (2008). Development of SCAR marker for authentication of *Pueraria tuberosa* (Roxb. ex. Willd.) DC. Current Science..

[35] Vassou SL, Kusuma G, Parani M (2015). DNA barcoding for species identification from dried and powdered plant parts: a case study with authentication of the raw drug market samples of *Sida cordifolia*. Gene..

[36] Liu C (2012). DNA barcode goes two-dimensions: DNA QR code web server. PloS One..

[37] Pang X (2010). Using DNA barcoding to identify species within Euphorbiaceae. Planta medica..

[38] Han, J. *et al*. The short ITS2 sequence serves as an efficient taxonomic sequence tag in comparison with the full-length ITS. *BioMed research international*. **2013** (2013). 10.1155/2013/741476 PMC3581084 23484151

[39] Čandek K, Kuntner M (2015). DNA barcoding gap: reliable species identification over morphological and geographical scales. Molecular Ecology Resources..

[40] Chen J (2015). Testing DNA barcodes in closely related species of *Curcuma* (Zingiberaceae) from Myanmar and China. Molecular ecology resources..

[41] Keller A (2010). Including RNA secondary structures improves accuracy and robustness in reconstruction of phylogenetic trees. Biology Direct..

[42] Gu W (2013). Application of the ITS2 region for barcoding medicinal plants of Selaginellaceae in Pteridophyta. PLoS One..

[43] Feng S (2016). Application of the ribosomal DNA ITS2 region of *Physalis* (Solanaceae): DNA barcoding and phylogenetic study. Frontiers in Plant Science..

[44] Mishra P (2016). DNA barcoding: an efficient tool to overcome authentication challenges in the herbal market. Plant biotechnology journal..

[45] Sucher NJ, Carles MC (2008). Genome-based approaches to the authentication of medicinal plants. Planta medica..

[46] Seethapathy GS (2015). Assessing product adulteration in natural health products for laxative yielding plants, *Cassia*, *Senna*, and *Chamaecrista*, in Southern India using DNA barcoding. International journal of legal medicine..

[47] Wu L (2015). An integrated system for identifying the hidden assassins in traditional medicines containing aristolochic acids. Scientific reports..

[48] Newmaster SG (2013). DNA barcoding detects contamination and substitution in North American herbal products. BMC medicine..

[49] Wang XY, Liu Y, Wang LL, Han JP, Chen SL (2016). A Nucleotide Signature for the Identification of Angelicae Sinensis Radix (Danggui) and Its Products. Scientific reports..

[50] DeSalle RO (2006). Species discovery versus species identification in DNA barcoding efforts: response to Rubinoff. Conservation Biology..

[51] Keller A (2009). 5.8 S-28S rRNA interaction and HMM-based ITS2 annotation. Gene..

[52] Nilsson RH (2012). Five simple guidelines for establishing basic authenticity and reliability of newly generated fungal ITS sequences. MycoKeys..

[53] Tamura K (2011). MEGA5: molecular evolutionary genetics analysis using maximum likelihood, evolutionary distance, and maximum parsimony methods. Molecular biology and evolution..

[54] Slabbinck B (2008). TaxonGap: a visualization tool for intra-and inter-species variation among individual biomarkers. Bioinformatics..

[55] Ross HA, Murugan S, Li WL (2008). Testing the reliability of genetic methods of species identification via simulation. Systematic biology..

[56] Gao T (2010). Identification of medicinal plants in the family Fabaceae using a potential DNA barcode ITS2. Journal of ethnopharmacology..

[57] Krawczyk K, Szczecińska M, Sawicki J (2014). Evaluation of 11 single-locus and seven multilocus DNA barcodes in *Lamium* L. (Lamiaceae). Molecular ecology resources..

[58] Koetschan C (2012). ITS2 database IV: interactive taxon sampling for internal transcribed spacer 2 based phylogenies. Molecular Phylogenetics and Evolution..

