# Supplementary material for: Barcode ITS2: a useful tool for identifying Trachelospermum jasminoides and a good monitor for medicine market
Source: Sci Rep. 2017 Jul 11;7:5037. doi: 10.1038/s41598-017-04674-w (PMC5506054; doi:10.1038/s41598-017-04674-w)
Supplement: Supplementary file 1 — Supplementary Information [file 41598_2017_4674_MOESM1_ESM.pdf]

Title: Barcode ITS2: a useful tool for identifying *Trachelospermum jasminoides* and a good monitor for medicine market

Authors: Ning Yu,[1,2†] Yu-long Wei,[1,2†] Xin Zhang,[1,2] Ning Zhu,[1,2] Yan-li Wang,[1,2] Yue Zhu,[1,2] Hai-ping Zhang,[1,2] Fen-mei Li,[1,2] Lan Yang,[1,2] Jia-qi Sun,[1,2] and Ai-dong Sun[1,2*]

SUPPLEMENTARY INFORMATION

Appendix S1. List of analyzed samples

Appendix S1 List of analyzed samples

Latin Name	Voucher NO.	GenBank NO.	Locality	Identification Result
T. jasminoides	LS001	KX998404	Anguo Medicine Market, Baoding, Hebei	Genuine
T. jasminoides	LS002	KX998405	Anguo Medicine Market, Baoding, Hebei	Genuine
T. jasminoides	LS003	KX998406	Anguo Medicine Market, Baoding, Hebei	Genuine
T. jasminoides	LS004	KX998407	Anguo Medicine Market, Baoding, Hebei	Genuine
T. jasminoides	LS005	KX998408	Anguo Medicine Market, Baoding, Hebei	Genuine
T. jasminoides	LS006	KX998504	Anguo Medicine Market, Baoding, Hebei	Fake
T. jasminoides	LS007	KX998409	Anguo Medicine Market, Baoding, Hebei	Genuine
T. jasminoides	LS008	KX998410	Anguo Medicine Market, Baoding, Hebei	Genuine
T. jasminoides	LS009	KX998411	Anguo Medicine Market, Baoding, Hebei	Genuine
T. jasminoides	LS010	KX998412	Anguo Medicine Market, Baoding, Hebei	Genuine
T. jasminoides	LS011	KX998413	Anguo Medicine Market, Baoding, Hebei	Genuine
T. jasminoides	LS012	KX998414	Drug store, Lou di, Hu nan	Genuine
T. jasminoides	LS013	KX998415	Drug store, Xiang tan, Hu nan	Genuine
T. jasminoides	LS014	KX998416	Drug store, Shao yang, Hu nan	Genuine
T. jasminoides	LS015	KX998417	Drug store, Sui ning, Hu nan	Genuine

T. jasminoides	LS016	KX998418	Drug store, Yan ji, Ji lin	Genuine
T. jasminoides	LS017	KX998419	Drug store, Ha er bin, Hei long jiang	Genuine
T. jasminoides	LS018	KX998420	Yuzhou Medicine Market, Yuzhou, Henan	Genuine
T. jasminoides	LS019	KX998421	Yuzhou Medicine Market, Yuzhou, Henan	Genuine
T. jasminoides	LS020	KX998422	Yuzhou Medicine Market, Yuzhou, Henan	Genuine
T. jasminoides	LS021	KX998423	Yuzhou Medicine Market, Yuzhou, Henan	Genuine
T. jasminoides	LS022	KX998424	Yuzhou Medicine Market, Yuzhou, Henan	Genuine
T. jasminoides	LS023	KX998425	Yuzhou Medicine Market, Yuzhou, Henan	Genuine
T. jasminoides	LS024	KX998426	Yuzhou Medicine Market, Yuzhou, Henan	Genuine
T. jasminoides	LS025	KX998427	Yuzhou Medicine Market, Yuzhou, Henan	Genuine
T. jasminoides	LS026	KX998428	Yuzhou Medicine Market, Yuzhou, Henan	Genuine
T. jasminoides	LS027	KX998429	Xi’an Medicine Market, Xi’an, Shanxi	Genuine
T. jasminoides	LS028	KX998430	Xi’an Medicine Market, Xi’an, Shanxi	Genuine
T. jasminoides	LS029	KX998431	Xi’an Medicine Market, Xi’an, Shanxi	Genuine
T. jasminoides	LS030	KX998432	Xi’an Medicine Market, Xi’an, Shanxi	Genuine
T. jasminoides	LS031	KX998433	Xi’an Medicine Market, Xi’an, Shanxi	Genuine
T. jasminoides	LS032	KX998434	Xi’an Medicine Market, Xi’an, Shanxi	Genuine

T. jasminoides	LS033	KX998435	Xi’an Medicine Market, Xi’an, Shanxi	Genuine
T. jasminoides	LS034	KX998436	Xi’an Medicine Market, Xi’an, Shanxi	Genuine
T. jasminoides	LS035	KX998437	Xi’an Medicine Market, Xi’an, Shanxi	Genuine
T. jasminoides	LS036	KX998438	Xi’an Medicine Market, Xi’an, Shanxi	Genuine
T. jasminoides	LS037	KX998439	Xi’an Medicine Market, Xi’an, Shanxi	Genuine
T. jasminoides	LS038	KX998440	Drug store, Cheng du, Si chuan	Genuine
T. jasminoides	LS039	KX998441	Drug store, Cheng du, Si chuan	Genuine
T. jasminoides	LS040	KX998442	Drug store, Cheng du, Si chuan	Genuine
T. jasminoides	LS041	KX998490	Drug store, Cheng du, Si chuan	Fake
T. jasminoides	LS042	KX998443	Bozhou Medicine Market, Bozhou, Anhui	Genuine
T. jasminoides	LS043	KX998444	Bozhou Medicine Market, Bozhou, Anhui	Genuine
T. jasminoides	LS044	KX998445	Bozhou Medicine Market, Bozhou, Anhui	Genuine
T. jasminoides	LS045	KX998446	Bozhou Medicine Market, Bozhou, Anhui	Genuine
T. jasminoides	LS046	KX998447	Bozhou Medicine Market, Bozhou, Anhui	Genuine
T. jasminoides	LS047	KX998448	Bozhou Medicine Market, Bozhou, Anhui	Genuine
T. jasminoides	LS048	KX998449	Drug store, Hubei	Genuine
T. jasminoides	LS049	KX998450	Drug store, Hubei	Genuine

T. jasminoides	LS050	KX998451	Drug store, Hubei	Genuine
T. jasminoides	LS051	KX998452	Drug store, Hubei	Genuine
T. jasminoides	LS052	KX998453	Drug store, Hubei	Genuine
T. jasminoides	LS053	KX998454	Drug store, Hubei	Genuine
T. jasminoides	LS054	KX998455	Drug store, Hubei	Genuine
T. jasminoides	LS055	KX998456	Drug store, Hubei	Genuine
T. jasminoides	LS056	KX998457	Drug store, Hubei	Genuine
T. jasminoides	LS057	KX998458	Drug store, Hubei	Genuine
T. jasminoides	LS058	KX998459	Drug store, Hubei	Genuine
T. jasminoides	LS059	KX998460	Drug store, Hubei	Genuine
T. jasminoides	LS060	KX998461	Drug store, Ping ding shan, He nan	Genuine
T. jasminoides	LS061	KX998462	Drug store, Zhou kou, He nan	Genuine
T. jasminoides	LS062	KX998463	Drug store, Wei hai, Shan dong	Genuine
T. jasminoides	LS063	KX998464	Drug store, Wei hai, Shan dong	Genuine
T. jasminoides	LS064	KX998465	Drug store, He ze, Shan dong	Genuine
T. jasminoides	LS065	KX998466	Drug store, Da lian, Liao ning	Genuine
T. jasminoides	LS066	KX998467	Drug store, Zhe jiang	Genuine

T. jasminoides	LS067	KX998468	Drug store, Beijing	Genuine
T. jasminoides	LS068	KX998469	Drug store, Beijing	Genuine
T. jasminoides	LS069	KX998470	Drug store, Beijing	Genuine
T. jasminoides	LS070	KX998471	Drug store, Beijing	Genuine
T. jasminoides	LS071	KX998472	Drug store, Beijing	Genuine
T. jasminoides	LS072	KX998473	Drug store, Henan	Genuine
T. jasminoides	LS073	KX998474	Drug store, Henan	Genuine
T. jasminoides	LS074	KX998475	Drug store, Henan	Genuine
T. jasminoides	LS075	KX998476	Drug store, Henan	Genuine
T. jasminoides	LS076	KX998477	Drug store, Henan	Genuine
T. jasminoides	LS077	KX998478	Drug store, Henan	Genuine
T. jasminoides	LS078	KX998479	Drug store, Henan	Genuine
T. jasminoides	LS079	KX998480	Drug store, Henan	Genuine
T. jasminoides	LS080	KX998481	Fresh leaf, Xiamen	Genuine
T. jasminoides	LS081	KX998482	Fresh leaf, Xiamen	Genuine
T. jasminoides	LS082	KX998483	Fresh leaf, Huaihua	Genuine
F. pumila	BL001	KX998484	Anguo Medicine Market, Baoding, Hebei	——

F. pumila	BL002	KX998485	Anguo Medicine Market, Baoding, Hebei	——
F. pumila	BL003	KX998486	Anguo Medicine Market, Baoding, Hebei	——
F. pumila	BL004	KX998487	Drug store, Anhui	——
F. pumila	BL005	KX998488	Bozhou Medicine Market, Bozhou, Anhui	——
F. pumila	BL006	KX998489	Fresh leaf, Nanning, Guangxi	——
F. tikoua	DG001	KX998491	Anguo Medicine Market, Baoding, Hebei	——
F. tikoua	DG002	KX998492	Anguo Medicine Market, Baoding, Hebei	——
F. tikoua	DG003	KX998493	Anguo Medicine Market, Baoding, Hebei	——
F. tikoua	DG004	KX998494	Fresh leaf, Xianmen	——
F. tikoua	DG005	KX998495	Cheng du, Si chuan	——
F. tikoua	DG006	KX998496	Bozhou Medicine Market, Bozhou, Anhui	——
F. tikoua	DG007	KX998497	Drug store, Hunan	——
F. tikoua	DG008	KX998498	Drug store, Hunan	——
E. fortunei	FF001	KX998499	Anguo Medicine Market, Baoding, Hebei	——
E. fortunei	FF002	KX99500	Cheng du, Si chuan	——
E. fortunei	FF003	KX99501	Drug store, Guizhou	——
E. fortunei	FF004	KX99502	Drug store, Guangxi	——

E. fortunei	FF005	KX99503	Fresh leaf, Nanning, Guangxi	——
T. jasminoides	——	KX302800.1	GenBank	——
T. jasminoides	——	KX302799.1	GenBank	——
T. jasminoides	——	KX302798.1	GenBank	——
T. jasminoides	——	KX302797.1	GenBank	——
T. jasminoides	——	KX302796.1	GenBank	——
T. jasminoides	——	KX302795.1	GenBank	——
T. jasminoides	——	KT898236.1	GenBank	——
T. jasminoides	——	AB610209.1	GenBank	——
T. jasminoides	——	JF708198.1	GenBank	——
T. jasminoides	——	FJ980308.1	GenBank	——
T. jasminoides	——	GQ434435.1	GenBank	——
T. jasminoides	——	GQ434434.1	GenBank	——
F. pumila	——	JQ773956.1	GenBank	——
F. pumila	——	JQ773955.1	GenBank	——
F. pumila	——	AY063580.1	GenBank	——
F. pumila	——	GQ434709.1	GenBank	——

F. pumila	——	GQ434708.1	GenBank	——
F. tikoua	——	EU091641.1	GenBank	——
F. tikoua	——	KF492774.1	GenBank	——
F. tikoua	——	JQ774008.1	GenBank	——
F. tikoua	——	JQ774007.1	GenBank	——
F. tikoua	——	JQ774006.1	GenBank	——
F. tikoua	——	JN117648.1	GenBank	——
E. fortunei	——	KC999835.1	GenBank	——
E. fortunei	——	KC999834.1	GenBank	——
E. fortunei	——	KC999833.1	GenBank	——
